# Melanopsin Mediates Retrograde Visual Signaling in the Retina

**DOI:** 10.1371/journal.pone.0042647

**Published:** 2012-08-03

**Authors:** Dao-Qi Zhang, Michael A. Belenky, Patricia J. Sollars, Gary E. Pickard, Douglas G. McMahon

**Affiliations:** 1 Eye Research Institute, Oakland University, Rochester, Michigan, United States of America; 2 Department of Cell and Animal Biology, Hebrew University of Jerusalem, Jerusalem, Israel; 3 School of Veterinary Medicine and Biomedical Sciences, University of Nebraska, Lincoln, Nebraska, United States of America; 4 Department of Ophthalmology and Visual Sciences, University of Nebraska Medical Center, Omaha, Nebraska, United States of America; 5 Department of Biological Sciences, Vanderbilt University, Nashville, Tennessee, United States of America; Dalhousie University, Canada

## Abstract

The canonical flow of visual signals proceeds from outer to inner retina (photoreceptors→bipolar cells→ganglion cells). However, melanopsin-expressing ganglion cells are photosensitive and functional sustained light signaling to retinal dopaminergic interneurons persists in the absence of rods and cones. Here we show that the sustained-type light response of retinal dopamine neurons requires melanopsin and that the response is mediated by AMPA-type glutamate receptors, defining a retrograde retinal visual signaling pathway that fully reverses the usual flow of light signals in retinal circuits.

## Introduction

Visual signals are transduced from light to neural signals by the rod and cone photoreceptors in the outer nuclear layer of the retina and are projected to the brain via the axons of retinal ganglion cells, originating in the inner-most layer of the retina. Interposed between transduction and projection, visual signals are processed by synaptic interactions of horizontal, bipolar and amacrine cells, the cell bodies of which reside in the retinal inner nuclear layer. While this radial flow of neural signals from outer retina, to inner retina, and then to the ganglion cell layer is considered the canonical order of visual signaling, there are also feedback signals at multiple levels within retinal circuits [1], [2]. The most central of these feedback circuits are the dopaminergic amacrine cells that receive synaptic input in the inner retina and project interplexiform process back to the outer retina [3]. Dopamine is a retinal neuromodulator that acts on photoreceptors as well as multiple neurons and synapses throughout the retina to reconfigure retinal circuits for light-adapted vision [4], [5].

In addition to the rod and cone photoreceptors of the outer retina, a subset of retinal ganglion cells are also photoreceptive. These cells express the photopigment melanopsin and are specialized for sustained signaling of light intensity, signaling to the brain's biological clock and contributing to the pupillary light reflex [6]. The participation of ganglion cell photoreceptors as a source of intra-retinal retrograde light signals to dopamine neurons has been implied by results showing that the temporal and spectral characteristics of sustained-type dopamine neuron light responses match those of ganglion cell photoreceptors and are mediated by a glutamatergic synaptic mechanism [7]. Here we have tested whether the melanopsin photopigment of ganglion cell photoreceptors is necessary for retrograde light responses in dopamine neurons, and have examined the involvement of post-synaptic glutamate receptors in this retrograde signaling circuit.

## Results

**Figure 1:**
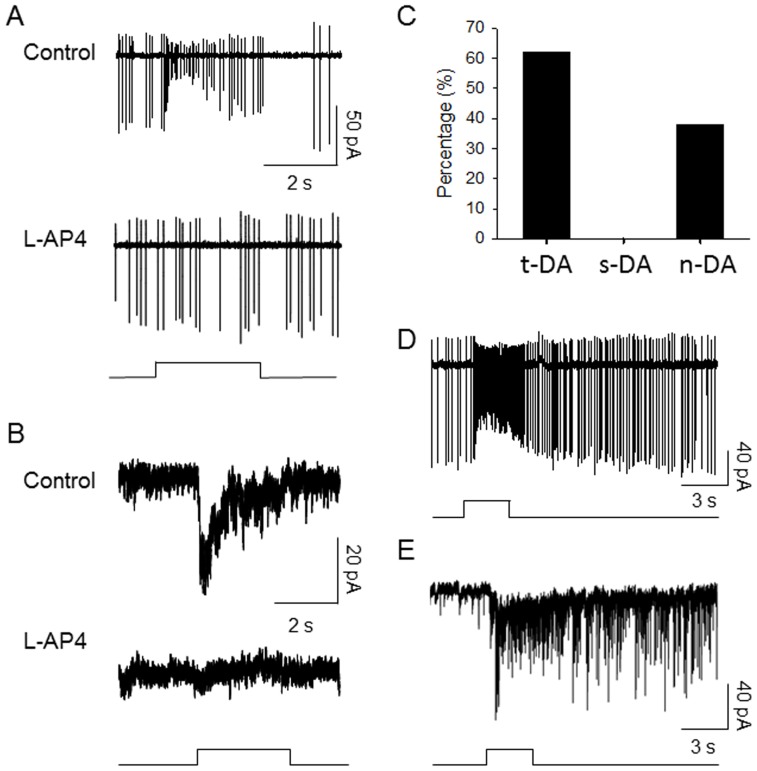
Transient-type light responses recorded from dopaminergic neurons in melanopsin knockout mouse retinas. (A) Loose patch recordings of action potentials, (B) whole cell recordings of light-induced inward currents. Bottom traces are recordings in the presence of 30 µM L-AP4 (C) Percentage of dopamine neurons exhibiting transient light responses (t-DA), sustained light responses (s-DA), and null light responses (n-DA). (D) Light-evoked spikes from a sustained-type dopamine neuron in a wild-type retina (n = 8). (E) Light-induced inward currents from a sustained-type dopamine neuron in a wild-type retina (n = 2). Stimulus bars indicate timing of 3 s pulses of 525 nm light in A, B (1.9×10^14^ photons cm^−2^ s^−1^) and 470 nm light in D and E (1.47×10^13^ photons cm^−2^ s^−1^).

To test the role of melanopsin in the light responses of retinal dopamine neurons, we crossed melanopsin knockout mice (OPN4^−/−^, [8]) with *TH*:RFP mice in which the retinal dopaminergic neurons are genetically labeled to facilitate targeting of these neurons for electrophysiology [9]. In mouse retinas wild-type for melanopsin, dopamine neurons exhibit distinct transient, sustained and null light responses with 38% of dopamine neurons exhibiting transient responses, 21% sustained responses, and 41% being unresponsive [10]. However, in OPN4^−/−^ retinas sustained light responses by dopamine neurons were completely absent, although a similar total proportion of dopamine neurons exhibited light responses (27/44, 61%, Fig. 1 A, B). Dopamine neurons in OPN4^−/−^ retinas exhibited null light responses to visible light, in similar proportion to the unresponsive neurons in wild-type animals (17/44, 39%, Fig. 1C). The loss of sustained light responses was due to the loss of both sustained light-induced spiking and membrane depolarization, as evident in loose-patch and whole-cell recordings of dopamine neurons (Fig. 1A, B). In OPN4*^−/−^* retinas all dopamine neuron light responses exhibited spike rate and light-induced inward currents with rapid onset and then decline back to baseline within ca. 1 s during light pulses, constituting transient responses as previously defined [7]. This is in contrast with the sustained-type light responses found in wild-type retinas that are maintained throughout the light pulse and for several seconds after light offset [7], examples of which are shown by loose patch extracellular recording (Fig. 1D) and whole-cell voltage-current clamp recording (Fig. 1E). Thus, while in wild-type retinas 21% of dopamine neurons exhibited sustained light responses none did so in OPN4^−/−^ retinas, a difference in proportion that is statistically significant (10/47 cells in wild type vs. 0/44 cells in OPN4^−/−^ mice, Fisher's exact probability test, p = 0.001). In addition, in melanopsin knockout retinas all dopamine neuron light responses were blocked by the mGluR6 receptor agonist L-AP4 (30 µM, 18/18 cells, Fig. 1 A,B), indicating that they were driven by rod or cone signaling through ON bipolar cells. These results directly complement findings from *rd1* rod/cone degenerate mouse retinas in which all light responses were found to be sustained and resistant to blockade by L-AP4, suggesting that they were driven by melanopsin ganglion cells and not by rod/cone transduction [7]. Our results here demonstrate that the sustained light responses of retinal dopamine neurons indeed depend on melanopsin.

**Figure 2:**
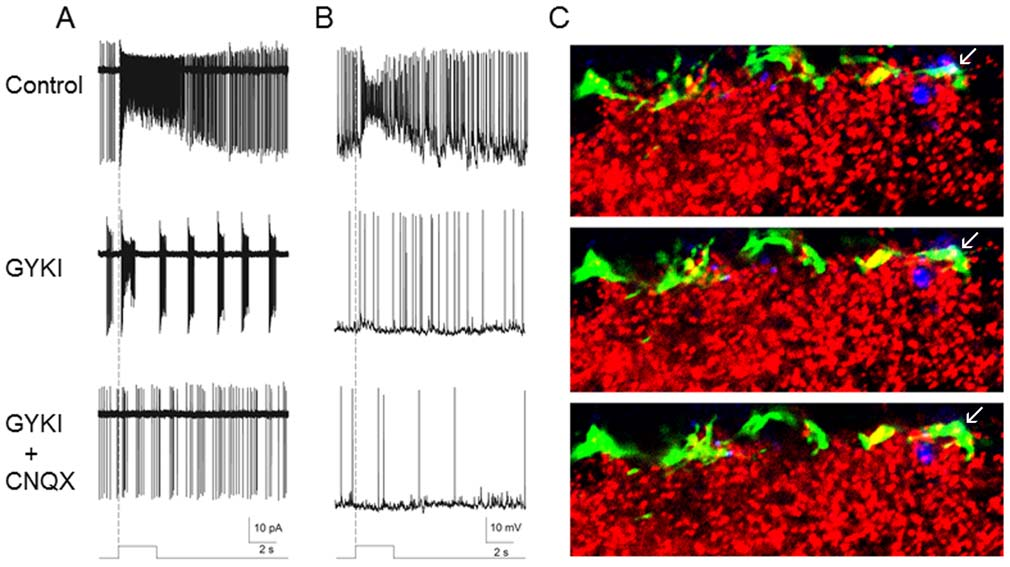
AMPA receptor mediation of synaptic transmission from melanopsin ganglion cells to dopamine amacrine cells in *rd1* rod/cone degenerate mouse retinas. Loose patch (A, n = 5) and whole cell (B, n = 3) recordings from dopamine amacrine cells showing substantial but incomplete blockade of light responses driven by melanopsin ganglion cells by application of the specific AMPA receptor blocker GYKI-52466 (200 µM, middle trace) and then complete block by co-application of the AMPA/kainite receptor blacker CNQX (100 µM, bottom). Stimulus bar shows the timing of the light pulse (470 nm, 3 s, 1.3×10^13^ photons cm^−2^ s^−1^), with the dashed vertical line showing light onset. (C) Confocal immunofluorescence of serial 0.5 µm optical sections of the retinal inner plexiform layer of a WT mouse showing TH staining for dopamine neuron processes (green), melanopsin staining for melanopsin ganglion cell processes (blue) and pan-AMPA receptor staining for AMPA receptor subunits GluR1–4 (red). AMPA receptors are present on dopamine cell processes (yellow), including at the apposition of melanopsin processes and dopamine cell processes (arrows).

Ganglion cell photoreceptors are glutamatergic and the sustained light responses of retinal dopamine neurons are driven by a glutamatergic mechanism [7]. To test the specific synaptic mechanism of retrograde transmission from ganglion cell photoreceptors to dopamine neurons we isolated melanopsin-driven responses by recording in retinas from *rd1* TH::RFP mice (6–8 months old) in which rods and cones had degenerated and then applied specific glutamate receptor blockers. Retrograde light-evoked spikes were substantially, but not completely, blocked by the specific AMPA-type glutamate receptor blocker GYKI-52466 (200 µM, Fig. 2A). Addition of the AMPA and kainate receptor blocker CNQX (100 µM) along with the GYKI then completely blocked retrograde light responses (n = 5). Consonant with its action on light-driven spikes, GYKI also substantially suppressed light-induced depolarization of dopamine neurons (Fig. 2B), and again co-application of CNQX gave complete suppression of light-induced depolarizing drive (n = 3). These results suggest that retrograde transmission from ganglion cell photoreceptors to dopamine neurons is mediated in large part by AMPA-type post-synaptic glutamate receptors, with the possibility of a smaller component of kainite receptor transmission.

To confirm the presence of AMPA-type glutamate receptors at sites of apposition of ganglion cell photoreceptors and retinal dopamine neuron processes in the inner plexiform layer, we performed triple-label immunocytochemistry for tyrosine hydoxylase (TH), melanopsin, and AMPA receptor subunits GluR1–4 in combination with confocal microscopy (Fig. 2C). We observed numerous sites of apposition between ganglion cell photoreceptor processes and retinal dopaminergic neurons in the inner plexiform layer near the border of the inner nuclear layer [11], [12], a subset of which contained AMPA-type glutamate receptor labeling on the TH+ process. These results are consistent with the AMPA-receptor mediated glutamatergic transmission from melanopsin ganglion cells to dopamine neurons shown by electrophysiology (Fig. 2A and B).

## Discussion

Our results establish that expression of melanopsin photopigment is necessary for the sustained-type light responses in retinal dopamine neurons that are not mediated by rod/cone photoreceptors. Since in the mammalian retina melanopsin is only known to be expressed in ganglion cells, this is strong evidence that the sustained light responses of dopamine neurons are driven by ganglion cell photoreceptors. A subset of retinal dopamine neurons therefore form an intra-retinal retrograde light signaling pathway where light transduction is initiated in the ganglion cell layer and is ultimately transmitted to the outer retina by dopaminergic transmission from interplexiform processes.

The synaptic mechanism for retrograde signaling from ganglion cell photoreceptors to dopamine neurons clearly involves AMPA-type glutamate receptors, with the likelihood of a modest contribution by kainite receptors as well. The localization of AMPA receptor proteins to dopamine neuron processes apposed to melanopsin-expressing ganglion cell processes supports the physiological role of AMPA receptors in this synaptic mechanism. Both dendrites and recurrent axon collaterals of ganglion cells have previously been shown to be presynaptic elements of synapses in the inner plexiform layer of the retina, establishing two potential routes by which retrograde transmission could take place [13]–[15]. A limitation of our present study is that while co-localization of AMPA receptors on dopamine neuron processes at sites of melanopsin ganglion cell apposition can be shown, the extent of these contacts cannot be accurately quantified with the methods used. To effectively visualize AMPA receptors in paraformaldehyde-fixed retinal tissue, we employed a pepsin pre-treatment as described by Fukaya et al., [16] which increased the intensity of AMPA immunostaining but at the same time diminished both TH and melanopsin immunostaining intensity, rendering the representation of melanopsin/AMPA/TH appositions in these experiments qualitative, rather than quantitative.

In the absence of melanopsin expression all recorded light responses in DA neurons are transient in time-course and blocked by inhibition of rod/cone transmission to ON bipolar cells by L-AP4. The proportion of DA cells responding to visible light in OPN4−/− retinas is identical to the total proportion of both transient and sustained light responses in wild-type retinas (ca. 60%). This is consistent with the notion that sustained DA neurons receive both ON bipolar and ipRGC input. This suggestion is further supported by our previous observations that sustained DA neuron responses exhibit a transient component that is blocked by inhibition of ON bipolar cell transmission [7], and anatomical studies suggesting that most DA neurons receive bipolar cell input [17]–[19].

What could be the function of the retrograde light signaling pathway in the retina? Ganglion cell photoreceptors are specialized for sustained signaling of overall luminance and their principal downstream targets are the brain's central biological clock and the circuit that drives the pupillary light reflex [6]. Thus by analogy their intra-retinal functions are likely to include circadian synchronization and sustained signaling of the overall luminance. It is therefore striking that they provide their sustained input to the dopaminergic amacrine cells that are a key retinal neuromodulatory system for light-adaptation and for synchronization and expression of retinal circadian rhythms [5], [20]. Indeed, retinal circadian rhythms are disrupted in melanopsin knockout mice and in retinal dopamine knockout mice [5], [21]. Therefore a single cell class, the ganglion cell photoreceptor, may provide both upstream retinal circuits and downstream brain circuits with sustained signals encoding luminance. With establishment of this novel retinal pathway it is apparent that visual signaling in the retina is a two-way street - with phototransduction initiated in the rod/cone photoreceptors driving ganglion cells through conventional retinal circuits, and photic signals originating in ganglion cell photoreceptors influencing outer retinal photoreceptors and neurons through a retrograde circuit that drives dopaminergic neurons.

## Materials and Methods

### Animals

Transgenic mice expressing red fluorescence protein (RFP) under the control of the tyrosine hydroxylase (TH) promoter were originally generated at Vanderbilt University [9] The mice were imported into the University of Nebraska, Lincoln where they were crossed with *OPN4^−/−^* mice (kindly provided by Drs. Samer Hattar and King-Wai Yau at Johns Hopkins University) in order to make *OPN4^−/−^ TH*::RFP transgenic mice. The new mouse line was sent back to and housed at Vanderbilt University for electrophysiology experiments. The *TH*::RFP transgenic mice were also imported to Oakland University where they were crossed with *retinal degeneration* 1 (Pde6b^rdl^) mice (purchased from the Jackson Laboratory) to make *rd1 TH*::RFP transgenic mice. In addition, C57BL/6 mice were used for confocal immunofluorescence . All animals were maintained under 12 h light: 12 h dark conditions. All procedures conformed to NIH guidelines for work with laboratory animals and were approved by the Institutional Animal Care and Use Committees at Vanderbilt University, Hebrew University of Jerusalem, University of Nebraska, Lincoln, and Oakland University.

It is worth noting that dopamine neurons remain morphologically intact in *rd1* retinas, but the level of expression of the RFP marker is decreased, significantly reducing the total number of marked cells that can be targeted for recording in rd1/TH::RFP retinas (Fig. S2 in [7]). In the present study, a total of 25 dopamine cells recorded in *rd1* retinas, 10 cells or 40% of them had light-evoked responses (all sustained).

### Electrophysiology

Mice were dark-adapted for 1–2 h prior to the experiments. They were then euthanized by CO_2_ overdose and cervical dislocation. Their eyes were enucleated and hemisected at the ora serata under infrared illumination. The cornea and lens were removed from the eyes in a Petri Dish filled with oxygenated extracellular solution, which contained (in mM): 125 NaCl, 2.5 KCl, 1 MgSO_4_, 2 CaCl_2_, 1.25 NaH_2_PO_4_, 20 glucose and 26 NaHCO_4_, and the retina was separated from the sclera. The retina was placed photoreceptor side down in the recording chamber that was mounted on the stage of an upright conventional fluorescence microscope (Axioscope or Axio Examiner ; Zeiss, Oberkochen, Germany) within a light-tight Faraday cage. Oxygenated extracellular medium (pH 7.4 with 95% O_2_ and 5% CO_2_) was continuously perfused into the recording chamber at a rate of approximately 2–3 ml/min, and the superfusate was kept at approximately 32–34°C by a temperature control unit (TC-344A, Warner Instruments, CT).

The retina was maintained in darkness for 1 h prior to recording. Cells and recording pipettes were viewed on either a video monitor coupled to a CCD camera (Princeton Instruments, Trenton, NJ) or a computer monitor coupled to a Digital camera (AxioCam, Zeiss, Oberkochen, Germany) mounted on the microscope. *TH*::RFP expressing cells were first identified by fluorescence microscopy using a rhodamine or DsRed filter set with a brief “snap-shot” of fluorescence excitation light (1–5 s). Then the identified cells and glass electrode were visualized using infrared differential interference contrast (IR-DIC) optics for patch-clamp recording.

Patch-clamp recordings were made from soma of RFP labeled dopamine neurons using 4–7 MΩ electrodes and signals were amplified using either Axopatch 1-D or Axopatch 200B amplifiers (Molecular Devices, Sunnyvale, California). The pipette solution for whole-cell voltage-clamp experiments contained (in mM): 118 Cs methanesulphonate, 12 CsCl, 5 EGTA, 0.5 CaCl_2_, 4 ATP, 0.3 GTP, 10 HEPES, adjusted to pH 7.3 with CsOH. For whole-cell current-clamp recordings, the standard internal solution contained (in mM): 125 K-gluconate, 10 KCl, 0.5 EGTA, and 10 HEPE, adjusted to pH 7.3 with KOH. In addition, pipette solution for loose-patch recordings contained 150 mM NaCl and 10 mM HEPES, adjusted to pH 7.4 with NaOH. Current and voltage stimuli were generated and data acquired via either Digidata 1322A or Digidata 1440A digitizers (Molecular Devices, Sunnyvale, California) using Clampex 10 software (Molecular Devices, Sunnyvale, California). The data were analyzed offline using Clampfit 10 software (Molecular Devices, Sunnyvale, California).

Tetrodotoxin (TTX), which was used to block action potentials in the whole-cell voltage-clamp recordings, and L-(+)-2–4-amino-4-phosphonobutyric acid (L-AP4) were purchased from Tocris Cookson (Ellisville, MO). All other drugs including 1-(4-Aminophenyl)-4-methyl-7,8-methylenedioxy-5H-2,3-benzodiazepine (GYKI-52466) hydrochloride and 6-cyano-7-nitroquinoxaline-2,3-dione disodium salt (CNQX) were purchased from Sigma Aldrich (St. Louis, MO). All drugs were prepared as concentrated stock solutions and diluted to working concentrations in extracellular medium.

Light stimuli were generated using a tri-color light-emitting diode (LED) lamp with 470-nm, 525-nm and 630-nm wavelengths (L.C. Corp, Brooklyn, NY). A LED controller (Mightex, Pleasanton, CA) was used to drive the LED and light intensity was adjusted by varying the driving current. The photon fluxes (photons cm^−2^ s^−1^) indicated in the figures were measured at the surface of the retina.

### Immunocytochemistry

Three different primary antibodies were used in the present study. AMPA receptors were detected using a pan-AMPA receptor antibody (GluRα1–4, 1∶150 ) raised in guinea pig against an amino acid sequence 717–754 of mouse GluRα1 that included a 19 amino acid sequence common to GluRα1–4 (Frontier Institute Co Ltd, Ishikari, Japan; [16]). Melanopsin was visualized using the UF006 antibody (1∶900) generated in rabbit (Covance Labs, Denver, PA) against the 15 N-terminus amino acid sequence of the predicted mouse protein [22]. A sheep antibody (1∶300) generated against tyrosine hydroxylase (Novus Biologicals, CO) was employed to label dopaminergic amacrine neurons.

Three adult mice (C57BL/6) were used for confocal immunofluorescence. Animals were perfused with freshly prepared 4% paraformaldehyde in 0.1 M phosphate buffer (pH 7.4). The eyes were dissected out and submerged into ice-cold fixative, retinas were carefully dissected and placed in the same fixative for an additional two to three hours. Retinal samples were embedded in 7% gelatin dissolved in water, treated overnight at 4°C with half-strength fixative, and sectioned at 40 µm with a Vibratome. Free floating sections stored in phosphate buffer saline (0.1 M PB containing 0.9% NaCl), were pre-heated for 30 min at 37°C and then treated for 10 min at the same temperature with 0.5 mg/ml pepsin diluted in 0.2 M HCl to expose AMPA antigen. The digestion was stopped with several changes of PBS. Samples were incubated in a blocking solution (2% egg albumin, 0.5% glycine, 0.5% lysine, in PBS, 1 h at room temperature) followed by a cocktail of primary antibodies diluted in PBS containing 1% egg albumin. Subsequently, 0.3% Triton X-100 was added both to the blocking solution and the primary antibody solution, and the samples were incubated for 48–72 h at room temperature. After thorough washing in PBS the retinal sections were incubated for 90 min at room temperature in secondary antibodies: donkey anti-sheep IgG conjugated with AlexaFluor 488 (1∶500; Molecular Probes, Eugene, OR), donkey anti-guinea pig IgG conjugated with Cy 3 and donkey anti-rabbit IgG conjugated with DyLight 649 (1∶300; both from Jackson ImmunoResearch Laboratory, West Grove, PA). Samples were mounted on subbed slides and coverslipped with Vectashield mounting medium (Vector Laboratories). Immunofluorescence material was examined using an Olympus FluoView 1000 confocal microscope. Sequential 0.5 µm optical sections were collected for green, red and blue channels, respectively, and are presented as single optical sections.
